# Circulating microRNAs - a new horizon in molecular diagnosis of breast cancer

**DOI:** 10.18632/genesandcancer.66

**Published:** 2015-05

**Authors:** Hoda A. Hagrass, Samar Sharaf, Heba F. Pasha, Enas A. Tantawy, Randa H. Mohamed, Rasha Kassem

**Affiliations:** ^1^ Clinical Pathology Department, Faculty of Medicine, Zagazig University, Egypt; ^2^ Medical Biochemistry Department, Faculty of Medicine, Zagazig University, Egypt; ^3^ Microbiology and Immunology Department, Faculty of Medicine, Zagazig University, Egypt; ^4^ Surgery Department, Faculty of Medicine, Zagazig University, Egypt

**Keywords:** Biomarker, breast cancer, microRNAs, real time PCR, diagnosis

## Abstract

**Background:**

The potential use of microRNAs (miRNAs) as ideal tumor markers has been the focus of recent research.

**Objective:**

Our hypothesis was that circulating miRNAs are differentially expressed in pretherapeutic sera of breast cancer patients compared to controls.

**Materials and Methods:**

Using real-time quantitative polymerase chain reaction (qPCR) analysis, levels of 5 candidate miRNAs (miR10b, miR34a, miR155, miR195 and miR16) were quantified in sera of breast cancer patients and control individuals.

**Results:**

Levels of preoperative sera showed significant upregulation of 3.36 fold rise in miR10b (*p*<0.001), a 2.07 fold rise in miR155 (*p* =0.005) and remarkable over expression of 11.9 fold rise in miR195 (*p*<0.001) of cases than controls. There was significant down regulation of miR34a (0.032, *p*<0.001). The comparison with the clinicopathological data of the breast cancer patients revealed significant high serum level of miR155 (*p* =0.004) and miR195 (*p* =0.002) in patients with lymph node metastasis and higher levels of miR10b (*p* =0.001) and miR155 (*p* <0.001) with distant metastasis (M1) than without metastasis (M0), in addition to significant decrease in miR34a (*p* <0.001) level in M1 than M0 cases.

**Conclusions:**

These findings suggest that systemic circulating miRNAs have potential use as novel biomarkers for breast cancer.

## INTRODUCTION

Breast cancer (BC) is the most common cancer and the second death causing cancer in women. Every year, more than one million women are diagnosed with breast cancer and approximately 400,000 die of it [1]. The disease is also considered the most common cancer in Egyptian women and accounts for 29% of National Cancer Institute cases. The age at diagnosis is lower than in countries of Europe and North America and most of the diagnosed females are premenopausal [2].

Early diagnosis of cancer remains a challenge for clinicians; it is an important goal to reduce treatment-associated morbidity & mortality and to reach maximal long-term survival [3].

MicroRNAs (miRNAs) are a class of small noncoding endogenous RNA molecules, 18–25 nucleotides long. Since their discovery, the investigators have found that they play important regulatory roles in many biological and pathological processes [4].

Tumors may be caused by down-regulation of a tumor suppressor miRNA and / or overexpression of an oncogenic miRNA. Micro RNA studies in breast cancer showed their importance and potential use in tumor classification and prognosis [5].

Iorio and colleagues found that miRNAs are deregulated in cancer tissues when compared with normal breast tissues and they emphasized the role of this deregulation in development of breast cancer [5].

As miRNAs regulate essential aspects of tumor biology and are stable in both blood and formalin-fixed, paraffin-embedded tissue samples, their use as potential prognostic and predictive markers has evolved [6,7].

Van Schooneveld and co-investigators profiled 768 miRNAs in 84 breast cancer tissue samples and 8 normal tissue samples obtained after breast-reductive surgery [8]. Whereas, Wang and colleagues showed that the high levels of miR9 and miR200c in breast cancers might serve as molecular diagnostic markers for the disease [9].

In this study, we aimed to investigate the utility of a panel of circulating miRNAs (miR10b, miR34a, miR155 and miR195) as potential biomarkers in breast cancer, particularly in early stages of the disease, this will be the first work studying these miRNAs in Egyptian female patients with breast cancer.

## RESULTS

One hundred and twenty breast cancer patients (48 premenopausal and 72 post menopausal) and 50 healthy controls were included in the analysis. Cases and controls were matched for age (mean age; 53±6.75 & 52±8.1years, for patients and controls respectively, *p*=0.16).

### Demographic and clinicopathological characteristics of breast cancer patients

All cases had histologically confirmed diagnosis (25 carcinoma in situ and 95 invasive carcinoma). Tumor grading revealed 53 cases of grades I &II and 67 cases of grade III. Based on TNM, 50 patients were in the early stages (0&I), while the other 70 patients were advanced (II, III and IV). Lymph node (LN) involvement was reported in 76 cases. Hormone receptors were evaluated in tissue samples: 80 cases were positive (+ve) for estrogen receptors (ER), 40 were ER negative (−ve), 83 were progesterone receptor (PR) +ve, 37 were PR−ve, 76 were HER2 –ve and 44 were HER2 +ve (Table 1).

**Table 1:** Demographic and clinicopathological characteristics of breast cancer patients

Variable	Frequency (%)
Age (53±6.75) years	
Premenopausal	48(40%)
Post menopausal	72(60%)
Types	
Carcinoma in situ	25 (20.8%)
Invasive carcinoma	95 (79.2%)
Grade	
I, II	53(44.2%)
III	67(55.8%)
Stage	
Early stage (0& I)	50(41.7%)
Advanced stage (II, III& IV)	70(58.3%)
ER	
−ve	40(33.3%)
+ve	80(66.7%)
PR	
−ve	37(30.8%)
+ve	83(69.2%)
HER2	
+ve	44(36.7%)
−ve	76(63.3%)
Lymph node	
−ve	76(63.3)
+ve	44 (36.7)

### Fold changes of miR16, miR10b, miR34a, miR155 and miR195 in breast cancer patients compared to the controls

As its expression is stable and reproducible, miR16 was chosen as an endogenous control to standardize miRNA expression. We quantified the relative expression of the four miRs (miR10b, miR34a, miR155 and miR195) in the blood of patients and controls. As shown in table 2, the analysis revealed a significant up-regulation in the form of 3.36 fold rise in miR10b (*p* <0.001), a 2.07 fold rise in miR155 (*p* =0.005) and a remarkable overexpression of 11.9 fold rise in miR195 (*p* <0.001) of cases than controls. In contrast, there was a significant down regulation of miR34a (0.032, *p* <0.001).

**Table 2:** Fold changes of miR16, miR10b, miR34a, miR155 and miR195 in Breast Cancer patients compared to the control group

	miR16	miR10b	miR34a	miR155	miR195
Fold change	1.11	3.36	0.032	2.07	11.9
95% CI	(0.68, 1.54)	(2.53, 4.19)	(0.02, 0.04)	(1.14, 3.01)	(6.17, 17.65)
p-value	0.63	<0.001*	<0.001*	0.005*	<0.001*

* Significant.

### Relationship between miRs (miR10b, miR34a, miR155 and miR195) in serum of breast cancer patients and their clinicopathological features

The relative concentrations of serum miRs (miR10b, miR34a, miR155 and miR195) of the 120 breast cancer patients were studied in relation to their clinicopathological data. The transcript levels of miR10b (*p*=0.001) and miR155 (*p*<0.001) were significantly higher in sera of patients with grade (III) than in those with lower grade tumors (I & II). Moreover, the serum level of miR155 was significantly increased in advanced stage cases (II, III& IV) than those in early stages (0 & I) (*p*=0.004). On the other hand, levels of miR34a were significantly down regulated in advanced stage (II, III& IV) cases than in early stages (0 & I) (*p*<0.001) (Table 3).

**Table 3:** Relationship between MicroRNAs’ (miR10b, miR34a, miR155 and miR195) fold changes and Clinicopathological Characteristics of patients

Parameters	miR10b	miR34a	miR155	miR195
Premenopause	3.1±0.9	0.038±0.026	1.85±0.17	11.8±7.8
Postmenopause	3.4±1.3	0.035±0.024	1.92±0.16	12.6±8.8
p-value	0.3	0.5	0.05	0.6
Type: Carcinoma in situ	2.98±0.9	0.035±0.026	1.85±0.16	10.8±3.6
Invasive carcinoma	3.4±1.2	0.037±0.025	1.9±0.18	12.7±9.2
p-value	0.1	0.8	0.2	0.3
Low grade (I, II)	2.9±0.8	0.039±0.031	1.83±0.19	13.2±9.98
High grade (III)	3.6±1.3	0.035±0.018	1.95±0.14	11.6±6.85
p-value	0.001*	0.4	<0.001*	0.3
Early stage (0 & I)	3.5±1.3	0.047±0.03	1.84±0.13	13.9±10.06
Advanced stage (II,III&IV)	3.1±1.04	0.028±0.017	1.93±0.19	11.1±6.77
p-value	0.07	<0.001*	0.004*	0.07
ER −ve +ve	3.8±1.2 3.1±1.1	0.032±0.021 0.038±0.027	1.85±0.22 1.91±0.15	13.5±10.2 11.7±7.3
p-value	0.001*	0.3	0.09	0.2
PR −ve +ve	3.2±1.1 3.3±1.1	0.031±0.021 0.038±0.026	1.88±02 1.89±0.16	12.6±10.9 12.1±7.1
p-value	0.6	0.2	0.8	0.8
HER2 −ve	3.1±1.1	0.036±0.032	1.90±0.18	13.7±11.4
+ve	3.4±1.2	0.036±0.02	1.88±0.17	11.5±5.4
p-value	0.3	0.98	0.5	0.1
Lymph node −ve	3.2±1.1	0.035±0.027	1.86±0.17	10.5±3.7
+ve	3.4±1.2	0.039±0.022	1.95±0.17	15.3±12.5
p-value	0.5	0.3	0.004*	0.002*
Distant metastasis M0	3.03±1.03	0.044±0.027	1.85±0.17	11.6±7.02
M1	3.8±1.2	0.023±0.012	1.96±0.16	13.5±10.4
p-value	0.001*	<0.001*	<0.001*	0.2

* Significant.

In respect to the relation between miRs serum levels and lymph node involvement, we found significantly higher serum levels of miR155 (*p* =0.004) and miR195 (*p* =0.002) in cases with lymph node metastasis compared to thoses with no lymph node metastasis (Table 3).

Remarkably, we revealed significantly higher serum levels of miR10b (*p* =0.001) and miR155 (*p* <0.001) in cases with distant metastasis (M1) than those with no distant metastasis (M0), in addition to a significant decrease in miR34a (*p* <0.001) serum levels in M1 than M0 cases (Table 3).

Regarding ER status, circulating miR10b levels were higher in ER −ve when compared with ER +ve patients (*p* =0.001), and no statistically significant difference was detected for other miRs.

The statistical analysis of the concentrations of the studied miRs in relation to the other clinicopathological features as well as the immunohistochemical presence of ER, PR & HER2 did not reach any statistical significance (Table 3).

## DISCUSSION

In this study, we analyzed the levels of four miRNAs (miR10b, miR34, miR155, and miR195) in serum from 120 BC patients and 50 healthy controls. Our assay showed significantly higher serum levels of miR-10b, miR155, and miR195 in BC patients and a significantly lower serum level of miR34 in BC patients.

The role of miR10b in breast cancer has been addressed with conflicting results. Earlier studies found that miR10b was down-regulated in breast tumors compared to normal breast tissues [5, 10]. Ma *et al*. and Mar-Aguilar *et al*. opposed these findings, and reported that miR10b was significantly higher in the serum of BC patients than controls which is in line with our results [11,12]. On the contrary, Heneghan and colleagues reported that the level of expression of miR10b in BC patients is similar to that of healthy controls [3].

With regard to miR34a, a discrepancy of its expression in diverse tumors has been described. Dutta and co-authors found a high incidence of miR34a overexpression in various tumor types, and undetectable expression in only poorly differentiated gastric adenocarcinomas and renal cell carcinomas [13]. In addition, Roth and colleagues showed high miR34a levels in the blood of breast cancer patients [14]. In contrast, it was reported that miR34a was downregulated in various cancer types including non-small cell lung carcinomas, pancreas tumor cell lines, colon carcinomas, primary neuroblastomas and breast cancer [15-17].

In the same line with our results, Mar-Aguilar *et al*. found that the levels of miR155 were significantly higher in serum of BC patients than in controls [12]. Up-regulation of miR155 has been observed in human primary breast cancers, and was over-expressed significantly in tumor specimens of BC [5, 18, 19]. Moreover, Wang and his colleagues reported that mi-R155b was consistently up regulated in cancer samples of both tissues and matching sera compared with their controls [20]. In contrary to our results, prior studies by Heneghan *et al*. and Zhu *et al*. reported no significant difference in the level of expression of miR 155 between BC patients and healthy controls [3, 21].

Concerning miR195, our results confirmed the results obtained by other studies. Zhang and his colleagues identified miR195 to be significantly elevated in breast tumors compared with normal breast tissues [22]. Also, Heneghan and colleagues demonstrated that miR195 is significantly increased in the blood of breast cancer patients in comparison to disease-free control subjects, and is able to discriminate breast cancer patients from healthy controls with high specificity and sensitivity [3].

Furthermore, we analyzed the correlation between miR10b, miR34a, miR155 and miR195 expression and the clinicopathological data. MiR10b and miR155 were significantly up regulated in higher malignancy grades compared with lower grade ones. Wang and co-authors reported that higher tumor grades may show significantly higher expression of miR155 [20]. Using primary breast cancer tissues and epithelial cells, Kong and colleagues observed that miR155 can play an important role in the transforming growth factor-β induced epithelial to mesenchymal transition (TGF-β-induced EMT), cell migration and cell invasion by targeting the small G-protein RhoA. They indicated that it might be a potential therapeutic target for breast cancer intervention [23].

miRNA34a was significantly down-regulated in advanced stages compared with early stages, while miR155 was significantly up-regulated in advanced stages compared with early stages of our patients. In contrast to our results, Roth and colleagues revealed high miR34a levels in the blood of patients with advanced tumor stages [14].

Furthermore, we observed a significant increase in serum miR10b and miR155 levels in metastatic breast cancer patients, compared with non-metastatic cases, while miR34a was significantly decreased.

Our results were in agreement with Roth and colleagues who found that the levels of miR10b significantly correlated with the occurrence of metastasis but not with Heneghan & his coworkers who observed a significant increase in tumor miR195 levels in metastatic breast cancers, and reported that this pattern was reflected in the circulation [14, 24]. On the contrary, Roth and colleagues detected high miR34a levels in the blood of metastatic breast cancer patients [14].

Supporting our results, Ma *et al*. reported that miR10b specifically played an important role in the metastatic process but not in primary tumor development. They found this miR to be highly expressed in metastatic breast cancer cells, and its overexpression initiated invasion and metastasis in a combination of mouse and human cell models by indirectly activating the prometastatic gene RhoC [11].

Our observation that circulating miR10b is higher in ER negative disease was keeping with the findings of previous studies [11, 20, 24] which reported that miR10b was highly correlated to the ER or PR expression; great differences were detected in miR expression levels in samples that are hormone receptor negative, this was a predictive factor for prognosis in patients with breast cancer.

Women with PR-positive tumors had higher miR155 expression than those with tumors that were PR negative [21]. In our study, we did not observe different serum miR155 levels between hormone receptor-positive and -negative patients.

A potential relationship between circulating miRNA levels, menopausal status, type of disease (in situ vs. invasive), LN and HER2/neu status was also investigated, but no statistically significant difference was identified for any of these parameters.

Results presented here showed significantly altered circulating levels of certain miRNAs (miR10b, miR34, miR155, and miR195) in breast cancer patients compared with healthy controls and a significant correlation between these miRNAs’ expression and some clinicopathological data. However, future larger studies and advanced technologies are needed to confirm our findings and to further explore the existing potential of circulating miRNAs as novel biomarkers for breast cancer in order to be utilized in the clinical field.

## MATERIALS AND METHODS

### Blood Collection and serum preparation

Blood samples were collected from 170 Egyptian female participants, including 120 consecutive breast, pathologicaly confirmed breast cancer patients admitted to Surgery Department of Zagazig University Hospitals (Zagazig, Egypt). Patients who had received chemotherapy or radiotherapy in the preoperative period were excluded. Fifty apparently healthy, age and sex-matched individuals, comprised the control group.

Three milliliters of venous blood were collected from the antecubital fossa and placed in a serum separator tube gel. The blood was centrifuged at 1600 rpm for 5 min and serum was transferred into 1.7 ml eppendorf tubes, then another centrifugation step was done at high speed 12,000 rpm for 15 min to remove cell debris completely, leaving only circulating RNA.

A written informed consent was obtained from all of the study participants.

### Tissue samples preparation

As regards tissue samples, four μm thick sections from formalin-fixed, paraffine-embedded tissue blocks were stained with hematoxylin-eosin for morphological assessment. Tumors were evaluated for tumor grading using the Elston and Ellis grading system for invasive carcinoma, and the criteria of the European Breast Screening Group for ductal carcinoma in situ (DCIS). Tumor staging was reported based on TNM, according to the World Health Organization (WHO 2003) classification of breast tumors [25, 26].

### Immunohistochemistry

Immunohistochemical staining was carried out using streptoavidin-biotin immunoperoxidase technique (Dako-cytomation, Glostrup, Denmark). Three of 5-μm thick sections cut from formalin fixed paraffin embedded blocks were stained for each sample, and scoring was performed independently by two different expert observers. Hormone receptors were evaluated in the tissue samples using monoclonal antibodies: anti-ER (mouse monoclonal IgG, code number sc-56833, Santa Cruz Biotechnology, CA), anti-PR (rabbit polyclonal IgG, code number sc-539, Santa Cruz Biotechnology, CA), anti-HER2 (mouse monoclonal IgG, code number sc-33684, Santa Cruz Biotechnology, CA), and secondary antibodies. Product visualization was then performed employing diaminobenzidine (DAB) substrate chromogen (DakoCytomation, Glostrup, Denmark).

### RNA Isolation

RNA Isolation was done according to the manufacturer's instructions by using the miRNeasy Mini Kit (QIAGEN GmbH, Hilden, Germany) that combines phenol/guanidine-based lysis of samples and silica-membrane–based purification of total RNA. QIAzol Lysis Reagent, included in the kit, is a monophasic solution of phenol and guanidine thiocyanate, designed to facilitate lysis of tissues, to inhibit RNases, and also to remove most of the cellular DNA and proteins from the lysate by organic extraction.

### Conversion of RNA into cDNA (complementary DNA)

RNA was reverse transcribed using miScript II RT Kit (QIAGEN GmbH, Hilden, Germany) according to the manufacturer's instructions. In each batch of reactions RT (reverse transcription) controls were included. Reverse transcription reactions were carried out in final volumes of 20 μl using an AmpGene DNA thermal cycler 4800. Briefly, reactions consisted of 15-μl RNA, 4-μl 5x miScript HiSpec Buffer containing nucleics mix and 1 μl miScript Reverse Transcriptase. Reactions were incubated for 60 min at 37°C then for 5 min at 95°C to inactivate miScript Reverse Transcriptase Mix. Finally, cDNA samples were Stored undiluted at −80°C.

### Analysis of miRNA Expression

We chose to study a panel of four miRNAs in the circulation of our cancer patients (miR10b, miR34a, miR155 and miR195) based on their previously documented associations with malignancies [4, 27] and miR16 was used as an endogenous control to detect the effect of hemolysis [28]. Expression level of miRNAs was analyzed using spiked-in Syn-cel-miR-39 miScript miRNA Mimic (QIAGEN GmbH, Hilden, Germany) was chosen and added as the normalized internal control.

Real-time PCR quantification of mature miRNA or noncoding RNA was performed using target-specific miScript Primer Assays (forward primers) and the miScript SYBR Green PCR Kit, which contains the miScript Universal Primer (reverse primer), and QuantiTect SYBR Green PCR Master Mix (QIAGEN GmbH, Hilden, Germany) according to the manufacturer's protocol.

Before Real-time PCR, cDNA samples (20 μl) were diluted by adding 100 μl PCR grade distilled water (DW). PCR reactions were carried out in final volumes of 25 μl using a StepOne™ System (Applied Biosystems). Briefly, reactions consisted of 2.5 μl cDNA, 12.5 μl 2x QuantiTect SYBR Green PCR Master Mix, 2.5 μl 10x miScript Universal Primer, 2.5 μl 10x miScript Primer Assay and 5 μl PCR grade DW.

Reactions were initiated with a 15-minute incubation at 95°C to activate HotStarTaq DNA Polymerase followed by 40 cycles at 94°C for 15 seconds (denaturation), 55°C for 30 seconds (annealing) and 70°C for 30 seconds (extension& fluorescence data collection). Each sample was duplicated and the mean of CT was calculated.

The relative expression of miRNA was calculated manually using the comparative cycle threshold (ßßCt) method as follows: ßCt = mean value Ct (miR of interest) - mean value Ct (reference miR), ßßCt= ßCT test sample − ßCT control sample.

The relative expression of miR of interest corresponded to the 2^(−ßßCt) value [29], normalized to spiked-in Syn-cel-miR-39 levels. Then the relative expression levels of miRNA were confirmed by using free data analysis tools at http://pcrdataanalysis.sabiosciences.com/mirna.

### Statistical analysis

Data was analyzed using SPSS statistical package version 17 (SPSS Inc., Chicago, IL). Independent *“t”* test was used for quantitative values. A *p*-value < 0.05 was considered significant.
